# Measuring resistance to externally induced movement of the wrist joint in chronic stroke patients using an objective hand-held dynamometer

**DOI:** 10.1016/j.cnp.2023.05.001

**Published:** 2023-05-16

**Authors:** Wala' Mahmoud, Morten Haugland, Ander Ramos-Murguialday, Hans Hultborn, Ulf Ziemann

**Affiliations:** a Institute for Clinical Psychology and Behavioral Neurobiology, University of Tübingen, Germany; b Department of Neurology & Stroke, University of Tübingen, Tübingen, Germany; c Hertie Institute for Clinical Brain Research, University of Tübingen, Tübingen, Germany; d Nordic Neurostim, Aalborg, Denmark; e Department of Neuroscience, University of Copenhagen, Denmark; f Tecnalia, Basque Research and Technology Alliance, San Sebastián, Spain; g Athenea Neuroclinics, San Sebastián, Spain

**Keywords:** Spasticity, Muscle stiffness, Spastic stroke, Spasticity measurement

## Abstract

**Objective:**

We evaluated the resistance to externally induced wrist extension in chronic stroke patients. We aimed to objectively measure and distinguish passive (muscle and soft tissue stiffness) and active (spasticity and spastic dystonia) components of the resistance.

**Methods:**

We used a hand-held dynamometer, which measures torque, joint movement and electromyography (EMG) simultaneously, to assess the resistance to externally induced wrist extension. Slow and fast stretches were applied to the affected and unaffected wrists in 57 chronic stroke patients (57 ± 11 years). We extracted from the data parameters that represent passive and muscle activity components and assessed the validity, test–retest reliability and the clinical utility of the measurement.

**Results:**

The analysis showed (1) a significant difference in the passive and muscle activity components between the affected and unaffected sides; (2) a significant correlation between passive and muscle activity components and the modified Ashworth scale (MAS); (3) a significant difference between the subgroups of patients stratified by the MAS; (4) an excellent intra-rater reliability on each of the passive and muscle activity components with intra-class coefficients between 0.92 and 0.99; (5) and small measurement error.

**Conclusions:**

Using a hand-held dynamometer, we were able to objectively measure the resistance to muscle stretch in the wrist joint in chronic stroke patients and discriminate muscle overactivity components from muscle and soft tissue stiffness. We demonstrated validity, test–retest reliability and the clinical utility of the measurement.

**Significance:**

Quantification of the different components of resistance to externally induced movement enables the objective evaluation of neurorehabilitation effects in chronic stroke patients.

## Highlights

• Hand held dynamometer enables objective measurement of resistance to externally induced movement in the wrist joint.
• Validity and reliability of the dynamometer makes it useful for evaluation of possible effects of rehabilitation interventions.
• Muscle overactivity components contribute significantly to resistance to externally induced movement in the wrist joint in stroke.

## 1 Introduction

An upper motor neuron injury due to stroke causes immediate paresis as well as a cascade of gradual changes that take place at the level of the brain, spinal cord, muscles and other soft tissues. These changes are triggered by the lesion itself as well as the immobilization and disuse of the paretic limb following the injury, for review see: Gracies, 2005a, Gracies, 2005b. A common consequence of these changes is the progressive development of abnormal response to muscle stretch in the paretic limb (Burke, 1988, Gracies, 2005b, Wissel et al., 2013). This feature is known as the primary hallmark of spasticity defined by Lance as “a velocity-dependent increase in tonic stretch reflexes (muscle tone) with exaggerated tendon jerks, resulting from hyperexcitability of the stretch reflex, as one component of the upper motor neuron syndrome” (Lance, 1980, Malhotra et al., 2009, Sommerfeld et al., 2012).

The increased resistance to muscle stretch that many subjects exhibit after stroke is a complex syndrome which includes both neurologic (spastic muscle overactivity), as well as non-neurologic (passive) soft tissue stiffness and muscle contracture components (Dietz and Sinkjaer, 2007, Lorentzen et al., 2010, Zhang et al., 2013). The assessment of this phenomenon is routinely performed during clinical neurological examination.

The neurologic (active) component which relates to spastic muscle overactivity includes two different components with distinct, but probably overlapping pathophysiological mechanisms. The first being *spasticity* which is the velocity-dependent increase in stretch reflex. For a given stretch velocity, stretch responses in spastic paresis are characterized by a decreased threshold and an increased amplitude compared to normal subjects (Galiana et al., 2005, Thilmann et al., 1991). The second is *spastic dystonia*, an involuntary static muscle contraction present in the absence of phasic stretch of the affected muscle (Forman et al., 2019, Gracies, 2005b, Marinelli et al., 2017). Spastic dystonia is, thus, the relative inability to relax muscles that is a constant feature in patients affected by spastic paresis (Nielsen et al., 2018).

The non-neurologic (passive) component contributes significantly to the increased resistance to muscle stretch (Vattanasilp et al., 2000) and emerges as an adaptation to muscle unloading and immobilization. The mechanisms underlying this adaptation include muscle atrophy (loss of muscle mass), loss of sarcomeres (loss of muscle length) and accumulation of intramuscular connective tissue and fat (reviewed by Gracies, 2005a).

Currently, the clinical evaluation of “spasticity” and the consequent therapeutic decisions are based mainly on physical examination, primarily using the (modified) Ashworth Scale (MAS), where an examiner moves a joint and simultaneously estimates the perceived resistance according to a 6-point ordinal scale that ranges between 0 (normal muscle tone) and 4 (rigidity) (Bohannon and Smith, 1986). MAS suffers from severe shortcomings in relation to its reliability and sensitivity, including poor intra- and inter-examiner reproducibility (Bhimani et al., 2011, Biering-Sørensen et al., 2006, Pandyan et al., 1999), a clustering effect in that most of the patients are grouped within the middle grades (Katz et al., 1992) and the outcome measure being a number that is based on subjective estimation by the examiner. Standardization, sensitivity, and objectivity are thus considered insufficient (Biering-Sørensen et al., 2006, Burridge et al., 2005, Lorentzen et al., 2010, Malhotra et al., 2008). More importantly, the MAS is unable to distinguish between the active (neurologic component) and the passive stiffness of the muscle (Fleuren et al., 2010). This has significant consequences for clinical decision-making, as many of the patients who have been clinically marked as “spastic” showed no signs of increased stretch reflex when they were investigated using electrophysiological and biomechanical tools (Sinkjær and Magnussen, 1994, Sinkjær et al., 1993, Toft et al., 1993). Administration of botulinum toxin, which inhibits the hyperexcitable stretch reflex, would not change the clinical picture nor improve the patients’ function in these cases (Alhusaini et al., 2011, Andringa et al., 2019a).

The different components underlying the spastic state might affect the functional disability of the individual patient and require treatment to a varying degree. Spasticity per se might not have a causal relationship to activity limitation. Indeed, the development of spastic muscle tone in the lower limb enables the patients to support their body weight during stepping and transfers (Dietz and Sinkjaer, 2007). However, combined with immobilization, some serious complications may arise if no treatment is provided. These include limitation and loss in range of motion, pain and contractures (Burridge et al., 2005).

In recent years, the potential of applying non-invasive brain stimulation paradigms to reduce post stroke spasticity has been explored. Indeed, there has been accumulating evidence for therapeutic utility of repetitive transcranial magnetic stimulation (rTMS) not only for improving motor function but also for reducing spasticity (Lefaucheur et al., 2020, McIntyre et al., 2018, Wang et al., 2022). In their recent systematic review and *meta*-analysis, Wang et al., (2022) concluded that the use of rTMS had a significant effect on reducing post stroke spasticity measured with the MAS. However, given the shortcomings of the clinical scale, establishing therapeutic effects on spasticity requires careful quantification of the reflex-mediated and other components of increased resistance to muscle stretching.

Several motor-based and hand-held devices have been developed to optimize the assessment of resistance to muscle stretch (Lindberg et al., 2011, Gäverth et al., 2013, Bar-On et al., 2013, Bar-On et al., 2014, Lorentzen et al., 2012, Yamaguchi et al., 2018). Some of these devices employ electromyography (EMG) to assess the stretch reflex, in combination with joint movement (kinematics) and applied torques (Bar-On et al., 2013, Bar-On et al., 2014, Lorentzen et al., 2012, Yamaguchi et al., 2018). This way the resistance to muscle stretch can be objectified and the neural and non-neural aspects discriminated (van den Noort et al., 2017). Biomechanical evaluation combined with electrophysiological measures might be considered a “gold standard” for combined measures of spasticity and contractures (Nielsen et al., 2018).

Only two devices are currently certified as medical devices and available on the market. The first is the Neuroflexor (Aggero MedTech, Alta, Sweden), a motorized device that extends the wrist and stretches the wrist and finger flexor muscles (Andringa et al., 2019b, Gäverth et al., 2013, Gäverth et al., 2014, Pennati et al., 2016). The second is the Portable Spasticity Assessment Device (PSAD, Movotec, Denmark), a hand-held dynamometer which integrates measurement of force, joint movement, and reflex-mediated muscle activity (Chen et al., 2020, Lorentzen et al., 2012, Svane et al., 2022, Willerslev-Olsen et al., 2013, Willerslev-Olsen et al., 2018, Yamaguchi et al., 2018). In a series of experiments, Yamaguchi et al. (2018) assessed the validity, accuracy and reproducibility of the measurement of passive and reflex-mediated stiffness in the ankle joint plantar flexors using the PSAD.

In another study, we investigate the effects of rTMS therapy on spasticity in chronic stroke subjects. In preparation for that study, we wanted to examine the validity of the use of the hand-held dynamometer to measure and distinguish components of increased resistance to muscle stretch in the wrist joint through assessment of (1) the difference between the affected and unaffected sides of stroke subjects; (2) the general agreement with the MAS; (3) the difference between subgroups of patients stratified by the MAS (clinical discrimination). We further examined the test–retest reliability as well as the measurement error and the smallest difference that indicates real change.

## 2 Materials and methods

### 2.1 Subjects

The study was performed on 57 patients (57 ± 11 years, 12 females) who had either a hemorrhagic or an ischemic stroke at least six months prior to the assessment (46 ± 53 months). All patients received TMS therapy in the TMS outpatient clinic at the Department of Neurology, Tübingen University Hospital, Germany. The clinical assessment was part of their clinical evaluation. Forty-six patients were assessed twice: before and after the rTMS intervention, and 23 patients had an additional follow-up measurement three months after the end of the intervention. In 45 patients, the measurements were performed on both the affected and unaffected sides. All patients gave their informed consent for the analysis and publication of their anonymized clinical data. The ethics committee of Tübingen University Hospital approved the study which complies with the 2013 update of the Declaration of Helsinki. Patients were excluded from the analysis only if when they suffered from severe contractures or severe limitation in the passive wrist range of motion which impeded the placement of the hand in the orthosis (n = 3). We did not set exclusion criteria with regard to the degree of resistance to muscle stretch, which meant that patients were included even if they exhibited flaccidity.

The Fugl-Meyer Assessment-upper extremity (FMA-UE) (Sanford et al., 1993) was used to evaluate motor disability, and patients were asked to fill in two questionnaires to capture their subjective experience. The first was a visual analogue scale where they reported, roughly, the degree of spasticity they perceived in their arm on a scale from 0 to 100. They also reported the difficulty they experienced in performing six activities of daily living due to stiffness in their affected side (Disability Rating Scale) (Bhakta et al., 2000, Platz et al., 2005). The patient group was heterogeneous with an FMA-UE mean score of 31 ± 16 (min = 0, max = 60). The self-evaluated spasticity level on the visual analog scale was 46 ± 25 (min = 0, max = 100). The individual scores are provided in Table 1.

**Table 1:** Characteristics of the patients included in the study.

ID	Age (years)	Sex	Time since stroke (months)	Affected side	FMA-UE/66	MAS wrist	Disability rating scale/24	Visual analog scale (%)
15	55	Male	38	Right	13	1	17	20
16	54	Male	14	Left	12	2	12	70
19	54	Male	51	Right	55	1	11	65
22	53	Female	22	Right	42	0	9	80
23	54	Male	32	Right	5	1	12	50
25	47	Male	54	Left	22	2	22	50
26	54	Male	37	Left	10	0	21	50
27	58	Male	35	Left	10	1+	18	0
28	74	Male	14	Right	18	2	14	60
29	38	Male	13	Left	14	1		30
30	51	Male	20	Left	42	0	14	50
31	51	Male	48	Left	26	0	13	75
32	44	Male	32	Left	11	2	3	30
33	57	Female	20	Left	41	0	11	0
34	36	Male	64	Right	28	1	14	
35	73	Male	8	Left	38	1	10	15
36	64	Male	45	Left	14	0	11	25
37	60	Male	19	Left	16	1	12	60
38	29	Female	27	Right	14	1+	11	80
39	48	Male	88	Left	9	3	21	100
41	27	Male	17	Left	18	2		60
42	52	Male	39	Right	48	1+	6	25
43	70	Male	43	Right	46	1+	12	50
44	70	Male	95	Right	7	3	19	65
45	49	Male	68	Left	19	1+	10	50
47	58	Female	80	Left	43	2	5	45
48	71	Male	40	Left	47	1+	11	35
49	64	Female	6	Left	32	2	12	50
1001	70	Female	40	Right	10	0	15	10
1004	62	Male	16	Right	22	1+	13	15
1005	50	Female	97	Left	49	1+	11	50
1007	69	Male	120	Right	39	1+	20	60
1009	54	Male	179	Left	58	1	8	40
1014	53	Male	45	Left	31	1	10	50
1023	54	Male	47	Right	28	2	7	60
1024	67	Male	50	Left	32	3	16	100
1025	60	Male	28	Left	44	1+	7	30
1032	39	Male	12	Right	38	2	17	33
1038	67	Male	14	Right	56	1	9	20
1039	49	Male	73	Right	38	3	4	75
1041	50	Female	14	Left	14	1	13	60
1018	70	Male	36	Left	57	0	5	40
1044	73	Male	71	Left	42	1+	11	30
1045	61	Male	73	Right	30	1	6	50
1047	53	Female	33	Left	41	0	12	70
1049	71	Male	9	Left	60	0	6	20
1055	58	Male	27	Right	56	0	7	0
1060	61	Male	35	Left	41	1	7	40
1061	66	Male	36	Right	34	1	12	55
1062	55	Male	14	Left	30	1+	4	30
1065	52	Male	29	Left	45	1	16	50
1073	60	Female	25	Left	59	0	7	60
1074	68	Male	128	Left	19	1+	11	90
1076	44	Female	12	Right	59	0	4	0
1077	59	Female	29	Right	21	2	20	30
1078	58	Male		Left	23	1+	8	40
1079	53	Female	178	Left	16	1	13	80

Abbreviations: ID: subject’s identification number; FMA-UE: Fugl-Meyer Assessment upper extremity (maximum score = 66); MAS: Modified Ashworth Scale (0–4). The disability rating scale is a questionnaire where the patients reported the difficulty they experienced in performing six activities of daily living due to stiffness in their affected side (0 no difficulty performing the task and 4 unable to perform it independently). The maximum score is 24 with higher scores representing more disability. The visual analogue scale is a questionnaire where patients reported the degree of spasticity they perceived in their arm on a scale from 0 to 100 (0 no spasticity at all and 100 the highest level of spasticity imaginable).

### 2.2 Assessment of resistance to externally induced movement

**Fig. 1:**
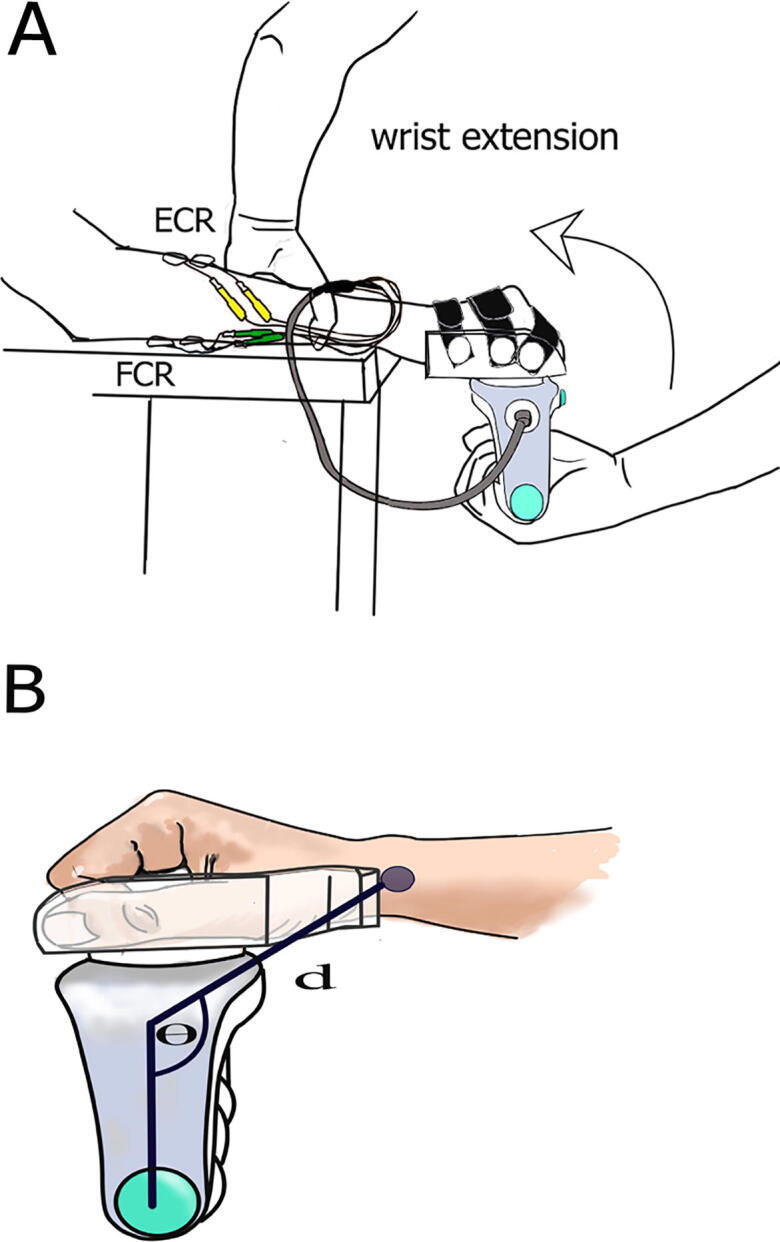
**Assessment set-up. A**: **set-up**: the orthosis is fixed to the patient’s hand using Velcro straps, and the device is attached to the orthosis. The EMG electrodes are placed on the Flexor Carpi Radialis (FCR-green) and Extensor Carpi Radialis (ECR-yellow) muscles. The experimenter fixes the patient’s forearm to the surface of the table while the wrist joint and hand rest outside the edge of the table. The experimenter holds the handle of the device and moves the wrist joint through the full available ROM (from full flexion to full extension) at two different velocities (fast and slow). **B**: Hand placement and position in reference to the device: the hand is fixed to the orthosis (transparent) by curling the fingers around the central knob. The finger joints are flexed between 45 and 60°and the thumb is slightly abducted and aligned with the medial surface of the index finger. Distance (d) and angle (θ) define the position of the device in reference to the center of rotation of the wrist joint. They are measured using a goniometer when the wrist joint is in a neutral position (180°). The filled circle represents the estimated center of rotation of the wrist joint. (For interpretation of the references to color in this figure legend, the reader is referred to the web version of this article.)

The subject was seated in an armless chair with the investigated arm placed on a height-adjustable table. The shoulder was slightly abducted, the elbow semi-flexed and the forearm pronated. In this position the wrist and hand extended slightly outside the edge of the table (Fig. 1A).

The Portable Spasticity Assessment Device (PSAD) is composed of a handle which encloses multiple sensors including dynamometers, accelerometers, and a gyroscope integrated with EMG. It enables the simultaneous assessment of muscle activity, torque and joint position. The hand held-dynamometer connects via WiFi to a data acquisition software installed on a laptop used for that purpose.

The handle of the device attaches to a custom-made plastic orthosis inside which the subject’s hand was fixed using padded Velcro straps in the “position of rest or function” (Savage, 1988). This position involves flexing the finger joints between 45 and 60°and placing the thumb in abduction and alignment with the medial surface of the index finger (see Fig. 1B). To allow this placement, the orthosis was designed to have a knob-like structure in its center, around which the fingers can curl. Placing the hand in this position prevents the stretching of the finger flexor muscles at the finger level, which would significantly limit the range of wrist extension due to passive tension in these muscles.

The axis of rotation of the wrist joint was defined as the virtual line which crosses the center of the wrist joint at the level of the ulnar styloid process (Fig. 1B). To measure the position of the device in relation to the wrist joint, we measured the distance (d), and angle (θ) between the center of mass of the device and the center of rotation of the wrist joint (Fig. 1B). The distance and angle were measured in a neutral wrist position (180°) and forearm pronation, and saved in the data acquisition software. In addition, the hand size (measured as the distance (cm) between the third knuckle and the middle of the wrist joint), orthosis size (small, medium or large), and height and weight of the subject were recorded and used for the optimization of the signal analysis.

EMG was recorded from both Flexor Carpi Radialis (FCR) and Extensor Carpi Radialis (ECR) muscles using bipolar surface adhesive electrodes (Covidien-Kendall, 24 mm) placed over the belly of the corresponding muscles with an interelectrode distance of 2 cm. The ground electrode was placed on the ulnar styloid process.

### 2.3 Range of motion assessment and fast and slow movements

Wrist extension range of motion (ROM) was recorded at the beginning of each measurement by moving the hand from full flexion (∼95°) until no further extension was possible (∼240°), by applying force to the wrist joint through the handle of the device. To assess the resistance to wrist extension, the experimenter moved the wrist joint passively through the full available extension ROM at two different velocities: the first is slow (below 20°/s) to assess the passive stiffness, ensuring that there is no triggering of a stretch reflex; and the other is fast (above 300°/s) to trigger a stretch reflex in the wrist flexors (Lewis et al., 2004, Lindberg et al., 2011). Throughout the duration of the assessment, the experimenter fixed the forearm of the subject to the surface of the table to ensure that all the utilized force is transferred through the handle of the device and is causing a rotation at the wrist joint only. This is essential for correct and consistent assessment the torque parameters.

A single measurement was composed of three slow and three fast stretches and we had two measurements for each side (6 slow and 6 fast stretches in total per side). During the performance of slow and fast stretches, the software provided online visual feedback on the velocity of the stretch the experimenter was applying. The experimenter could clearly see if they were moving the patient’s joint with the required velocity and whether the stretch was accepted by the software. An accepted stretch is one where the experimenter moved the joint with the required velocity, throughout 95% of the measured ROM. Patients were encouraged to relax completely and to avoid actively helping or resisting the movement. Visual feedback on activity in both wrist flexor and extensors muscles was provided in the form of a volume bar. When visible EMG activity was detected before the start of a stretch or during slow stretches, the measurement was rejected and repeated after encouraging and helping the patient to relax.

The position, acceleration, angular velocity and EMG data were sampled at a frequency of 1 kHz, while forces were sampled at a frequency of 500 Hz. All acquired data was saved for offline analysis. In accordance with the recommendations of the European Consensus on the Concepts and Measurements of the Pathophysiological Neuromuscular Responses to Passive Muscle Stretch (van den Noort et al., 2017), we aimed to extract passive stiffness and passive resistance from the slow stretch data, and active (reflex-mediated) resistance from the fast stretch data. For both velocities, we calculated average EMG activity, average work and the slope of the linear function fitted to torque–angle data.

### 2.4 PSAD signal analysis

A customized MATLAB code (version 2018b) was used to analyze the raw data based on the methods initially described in Yamaguchi et al., (2018) and modified for the wrist joint as follows. The gravitational torque of the hand was calculated using the estimated weight of the hand (0.065% and 0.05% of the total body mass for males and females respectively). The gravitational torque of the device was calculated based on the known weight and dimensions of the device and the attached orthosis. The distance (d) and angle (θ), which define the position of the hand in relation to the device (Fig. 1B) were used to calculate the lever arm of the hand (Fig. 1B). The analysis started by examining the recorded velocity signal and identifying slow and fast stretches**.** An example of the raw data is provided in the Appendix (Fig. A1.1).

### 2.5 Extracting parameters from slow and fast stretches:

#### 2.5.1 Slow stretches

Slow stretches are those where the movement velocity ranges between 0 and 20°/s. These are the trials where no stretch reflex could be evoked and where the torque applied could be accounted for by the stiffness of the muscle and soft tissues.

**Fig. 2:**
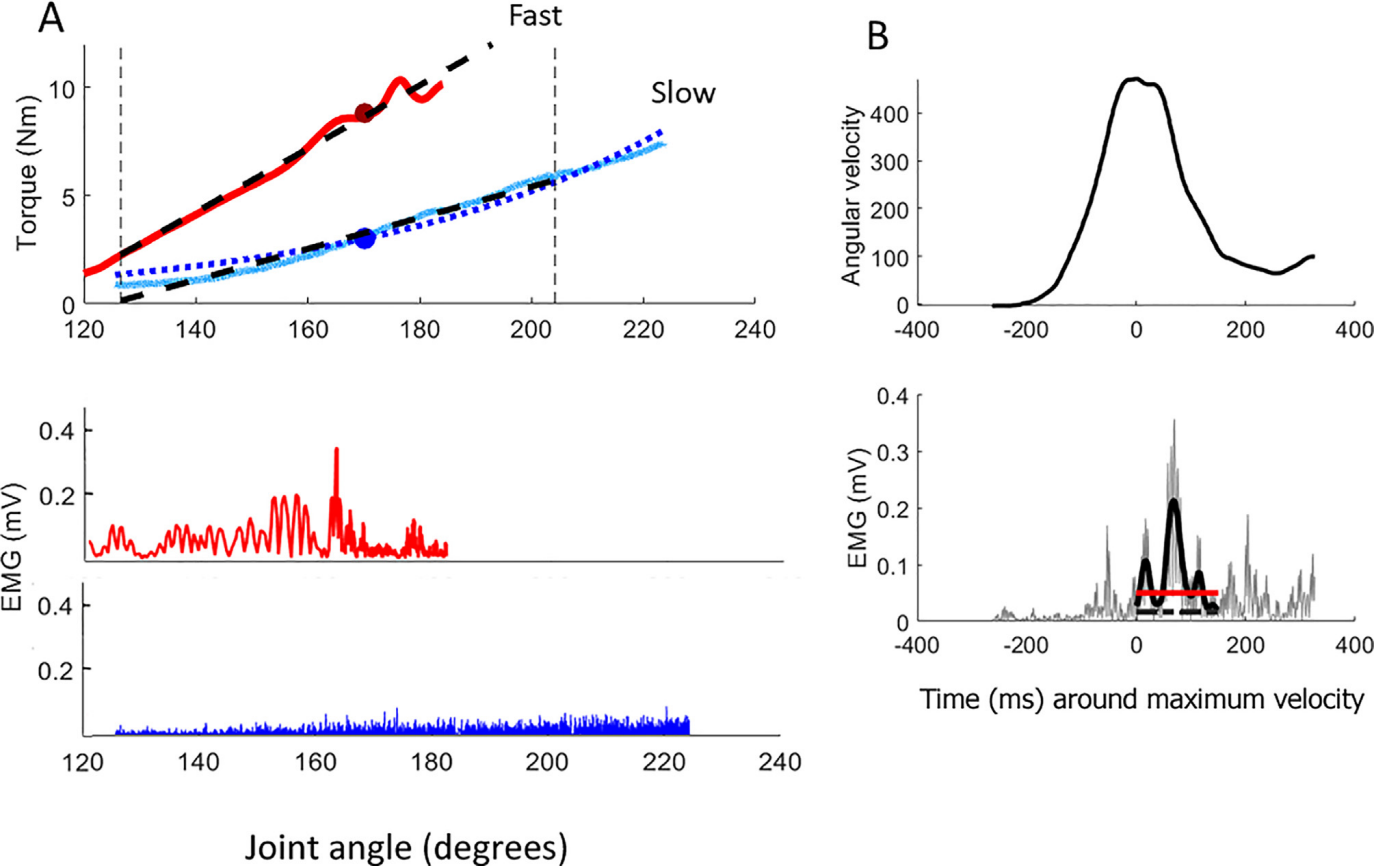
**Extracting outcome parameters from PSAD data**. **A:** The **upper panel** shows the torque data expressed as a function of the joint angle of one slow (blue) and one fast (red) stretch. The vertical dashed lines mark the middle 20–80% of the range of motion (ROM) used for the analysis. The dotted blue line represents the exponential function fitted to the slow torque data to allow measurement of the torque at any point of the ROM. The black dashed lines represent the linear function fitted to the slow and fast torque data. The red circle on the fast torque data trace represents the point in the ROM where the stretch reflex was detected (where we calculated the total resistance). The blue circle on the slow stretch torque trace is the equivalent point in the ROM during slow movement to that where the stretch reflex was detected during fast movements. This torque amplitude is the passive resistance which is subtracted from the total resistance (torque amplitude at the red circle) to calculate active (reflex-induced) resistance. The **lower panel** shows the FCR EMG activity during slow (blue MEG trace) and fast (red EMG trace). Abbreviations: Nm: Newton meter; mV: millivolt. **B: EMG peak detection (same dataset as in A)** for extracting reflex-mediated torque values. The upper panel shows the angular velocity curve function (black bell-shaped curve) in reference to time, where time 0 is the point of maximum velocity. The lower panel shows the detection of the stretch reflex. The gray EMG trace is the rectified EMG signal, while the thick black trace is the smoothed EMG signal within a window of 100 ms following maximum velocity. The amplitude of the smoothed EMG is compared to baseline activity (dashed black line). To count as a stretch reflex, the smoothed EMG peak within that window has to be at least 50 ÂµV in amplitude and to cross the threshold (red horizontal line) of 3 standard deviations of the background EMG activity. The torque (total resistance) will then be calculated in the window of 0–100 ms that follow the EMG peak. Abbreviations: mV: millivolt; ms: milliseconds. (For interpretation of the references to color in this figure legend, the reader is referred to the web version of this article.)

An exponential function was fitted to the torque data (expressed as function of angular displacement) to enable the estimation of the passive muscle stiffness at any given part of the range of motion (ROM) (Fig. 2A). We selected three positions corresponding to 30, 40, and 50% of the ROM for the extraction of passive stiffness values. **Passive stiffness** is defined as the change in torque required to move the joint 1°, averaged over the range from 5°below to 5°above these selected positions. The positions were selected because the stiffness measured there is mainly attributed to the muscle and minimally influenced by tendon and joint stiffness (Yamaguchi et al., 2018). The three resulting values were then averaged to obtain one representative measure of passive stiffness per stretch.

#### 2.5.2 Fast stretches

Fast stretches are those where the velocity of movement exceeds 300°/s. This velocity is considered sufficient for triggering a stretch reflex which is detectable in the EMG signal. The EMG signal was rectified, smoothed and filtered using a third order Butterworth band pass filter with the limits 75–175 Hz. To calculate baseline EMG activity, we ran a moving average over the whole EMG file and extracted the period which contained the signal with the lowest EMG amplitude that was stable for 3 s. The peaks of the EMG signal in the FCR muscle were identified using a customized peak detection function in MATLAB. Only the peaks within a window of 100 ms following the time of maximal velocity of the fast stretch were considered relevant reflex activity (Fig. 2B). We compared the smoothed EMG in the window of interest (100 ms after maximum velocity) to baseline EMG. For an EMG peak to be considered a valid stretch reflex response, its amplitude had to be ≥50 µV, and to be larger than 3 standard deviations of the baseline EMG. The 50 µV threshold was set to avoid accepting EMG peaks with low amplitudes in files with very low baseline activity. The number of valid stretch reflexes per measurement was extracted as an outcome parameter.

#### 2.5.3 Total resistance

The EMG-torque rise time for the FCR muscle is equivalent to ≈0–85 ms after EMG peak (Cavanagh and Komi, 1979). We thus considered the window of 100 ms after the onset of the stretch reflex (interval 0–100 ms after the EMG peak) to calculate the average torque which includes both active and passive elements and is defined as the total resistance. When no stretch reflex was detected, we averaged the torque in the 100 ms window that surrounds the time of highest angular velocity. To calculate the active (reflex-mediated) r**esistance**, we worked out the torque amplitude in the window where the total stiffness was calculated (0–100 ms after the EMG peak). The exponential function fitted to the slow stretch data (dotted line in Fig. 2A) was used to extract this torque value, **passive resistance**. The reflex-mediated stiffness was then calculated by subtracting the measured or estimated passive resistance from the calculated total stiffness. To examine the relative contributions of the passive and reflex mediated components to the total measured resistance, we also calculated the ratio of passive resistance to total resistance during fast stretches for each subject.

#### 2.5.4 Slope of the torque–angle linear function for fast and slow stretches

The previously described parameters (passive stiffness and passive and active resistance) are all based on points or very small parts of the ROM. In order to describe the development of torque during the whole movement, we fit a linear function to the torque data between 20% and 80% of the ROM. We report the coefficient of the linear function (slope) for slow and fast trials.

#### 2.5.5 Average work (Nm)

Work is the product of multiplying torque by the angular change (in degrees). It is also considered a more holistic outcome parameter. To calculate work, we split the mid 20–80% of the ROM data into 20 bins with each bin covering 3% of the ROM, and averaged the torque value for each bin. Work was then calculated for each individual bin, then averaged all 20 bins to get a single value for each slow and fast trial.

## 3 Statistical analysis

All the extracted parameters (passive stiffness, passive resistance, active resistance, average work (slow/fast), slope of the torque angle linear function (slow/fast), and average EMG (slow/fast) were transferred to IBM-SPSS Statistics software version 28.0.1.1 (IBM Corp., Armonk, NY, USA) for analysis. For the reliability analysis, we averaged the values extracted from one measurement (three slow and three fast stretches) to get a single value per measurement for each parameter. For all other tests, we averaged the values extracted from both measurements (6 slow and 6 fast stretches). To increase the statistical power, we included all the performed measurements per individual including those before and after the intervention.

### 3.1 Validity, reliability and clinical differentiation analyses

To examine the device’s ability to differentiate between the affected and unaffected sides, a paired samples *t*-test was run to compare the parameters measured from both sides. The general agreement with the MAS was explored by running a rank-based (Spearman) correlation analysis between each of the parameters and the MAS. For this analysis, only the measures extracted from the affected side were considered.

In order to test the device’s ability to differentiate between patient groups stratified by the MAS (clinical differentiation), we ran a one-way analysis of variance (ANOVA) with a post hoc analysis to test the effect of (GROUP) i.e., 5 different MAS scores (MAS 0, 1, 1+,2,3) on the different parameters extracted from the device. Since the equality of variances assumption was not met, the Games-Howell method for correction for multiple comparisons was used. A similar statistical analysis was run to test whether the contribution of the passive stiffness to the total measured stiffness (percentage) varied significantly across the different MAS scores. For the test–retest reliability analysis, we investigated the agreement between the values extracted from the two independent measurements performed by the same experimenter during a single measurement session. An intraclass correlation coefficient (ICC) analysis was run using a two-way mixed effects model for absolute agreement with 95% confidence interval (Trevethan, 2017).

### 3.2 Measurement error

The Intraclass Correlation Coefficients (r) and the standard deviations (for each of the two measurements) calculated in the repeatability analysis, were used to determine the measurement error according to the following formula:

$\mathrm{SEm}={\;}_{p}SD\surd \left(1-r\right)$where SEm is the standardized error of measurement, _p_SD is the pooled standard deviation and r is the correlation coefficient that results from the intraclass correlation coefficient analysis.

The pooled standard deviation is calculated as follows:

${\;}_{p}SD\surd \left(\left({SDm1}^{2}\right)+\left({SDm2}^{2}\right)\right)/2$where _p_SD is the pooled standard deviation, SDm1 is the standard deviation of the first measurement (m1), and SDm2 is the standard deviation of the second measurement (m2).

### 3.3 Minimal detectable change (MDC)

Minimal detectable change at the 68% and 95% confidence interval levels were then calculated as follows (Schuck and Zwingmann, 2003):

MDC68=SEm∗√2

where MDC68 is the Minimal detectable change at 68% confidence interval level, SEm is the standardized error of measurement.

MDC95=SEm∗1.96

where MDC95 is the minimal detectable change at 95% confidence interval level, Sem is the standardizes error of measurement.

#### 3.3.1 Principal factor analysis

In order to reduce the number of outcome parameters extracted from the PSAD data in the future, which might be necessary for increasing the clinical applicability of the method, we ran a principal factor analysis which yielded the smallest number of factors that describe the maximum percentage of variability in the data. The rationale, methods, results and conclusion of this analysis are available in Appendix A2.

## 4 Results

### 4.1 Differentiating affected and unaffected sides

**Fig. 3:**
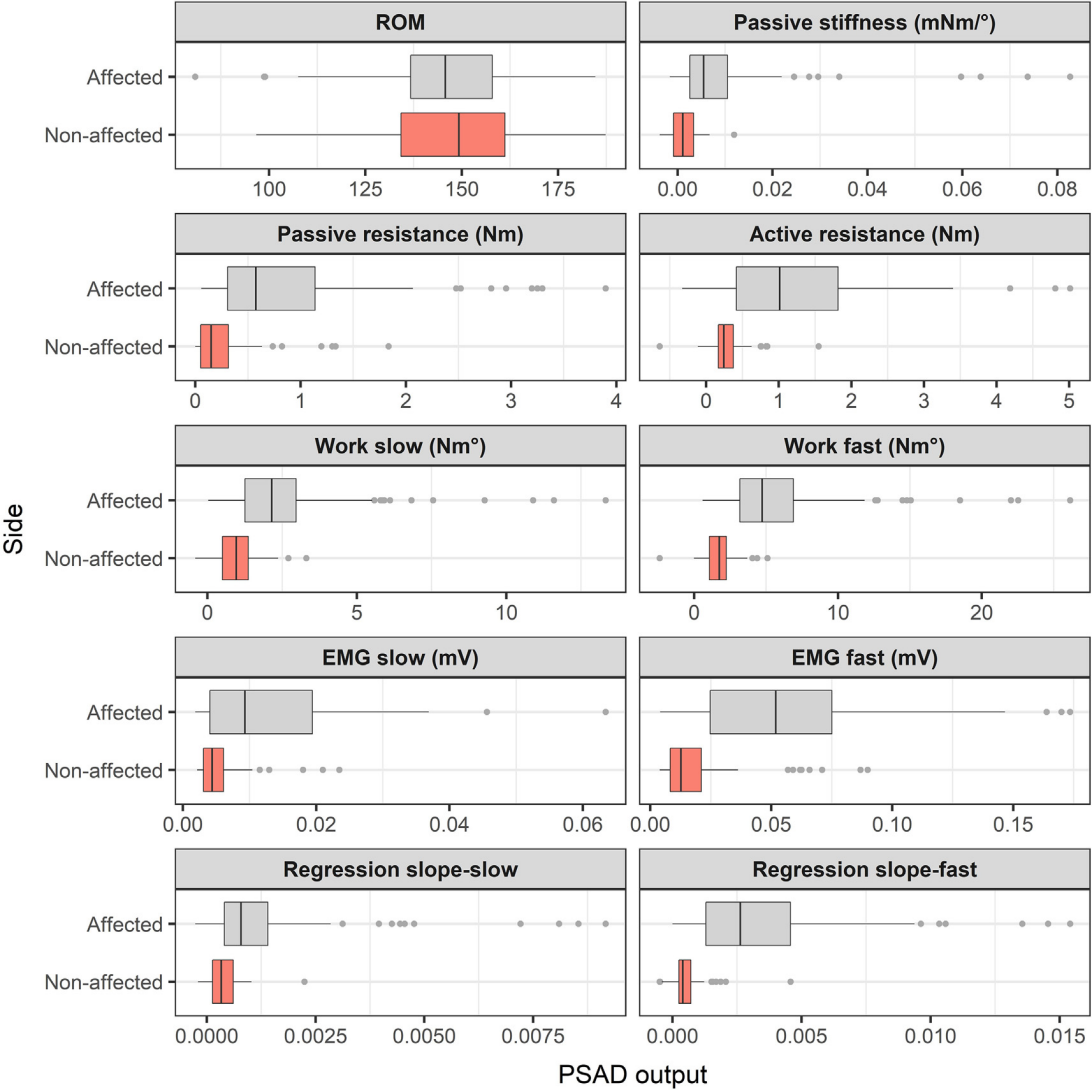
**The extracted PSAD parameters in the affected and unaffected sides.** The box plots (gray: affected and pink: unaffected) include all data from the affected vs. unaffected sides of all the stroke patients. Each parameter is represented in a different panel. The grey circles are outliers. Abbreviations: PSAD: portable spasticity assessment device; ROM: range of motion; mNm/°: milliNewton-meter per degree; Nm: Newtonmeter; Nm°: Newtonmeter.degree; mV: millivolt. (For interpretation of the references to color in this figure legend, the reader is referred to the web version of this article.)

The first step to establishing the validity of the application of the PSAD to the wrist would be to test its ability to distinguish between the affected and unaffected sides of the same subject. The same parameters were extracted from both sides and the values compared using a *t*-test. Fig. 3 shows the box plot distribution of data collected from the affected and unaffected sides for each of the extracted parameters.

The paired samples t-tests (Bonferroni-corrected for multiple comparisons) revealed a significant difference between the affected and unaffected sides on each of the measured parameters except the range of motion (ROM) (Fig. 3). Mean ROM was 147.7 ± 17° on the affected side, and 147.2 ± 17° on the unaffected side, with no statistical difference between the sides (df = 79, p = 0.79). The *t* statistic, degrees of freedom and significance for the other measured parameters were as follows: EMG for slow stretches (t_1,78_ = 5.6, p <.001), fast stretches (t_1,78_ = 7.9, p <.001), passive resistance (t_1,78_ = 3.9, p <.001), passive stiffness (t_1,78_ = 4.7, p <.001), active resistance (t_1,78_ = 3.9, p <.001), average work-slow (t_1,78_ = 7.3, p <.001), average work-fast (t_1,78_ = 4.7, p <.001), linear regression slope-slow (t_1,78_ = 6.5, p <.001), linear regression slope-fast (t_1,78_ = 4.1, p <.001).

Since the MAS score 0 refers to “no increase in muscle tone”, we were interested in finding out whether there was any difference between the outcome parameters recorded from the unaffected side and the affected side of patients who scored 0 on the MAS. The one-way ANOVA showed a significant effect for GROUP (unaffected side vs. affected side with MAS = 0) on the following parameters: EMG slow (F_1,117_ = 4.2, p =.04); work slow (F_1,117_ = 14.3, p <.001); EMG fast (F_1,117_ = 6.0, p =.016); passive stiffness (F_1,117_ = 7.0, p =.009); passive resistance (F_1,117_ = 4.5, p =.035). There was no difference in the following parameters: work fast (F_1,117_ = 3.2, p =.08); active resistance (F_1,117_ = 0.05, p =.83) and slope of the torque angle function for both velocities.

### 4.2 General agreement with the modified Ashworth scale (MAS)

In order to test the device’s agreement with the most widely used clinical scale, we grouped the patients according to their score on the MAS (wrist extension) and explored the correlation between the different PSAD parameters and the MAS score. The Spearman rank-based correlation analysis showed a significant correlation between each of the parameters extracted from PSAD data and the MAS, see correlation values in Table 2 for 126 measurements.

**Table 2:** Correlation between PSAD parameters and the MAS**.**

Parameter	Correlation coefficient (r)	Significance (p)
Work slow	0.521	<0.001
Work fast	0.660	<0.001
Linear coefficient (slope) slow	0.487	<0.001
Linear coefficient (slope) fast	0.633	<0.001
Passive stiffness	0.493	<0.001
Active resistance	0.630	<0.001
EMG fast	0.680	<0.001
EMG slow	0.431	<0.001

PSAD: portable spasticity assessment device, MAS: modified Ashworth scale.

### 4.3 Clinical differentiation (between the MAS scores)

**Fig. 4:**
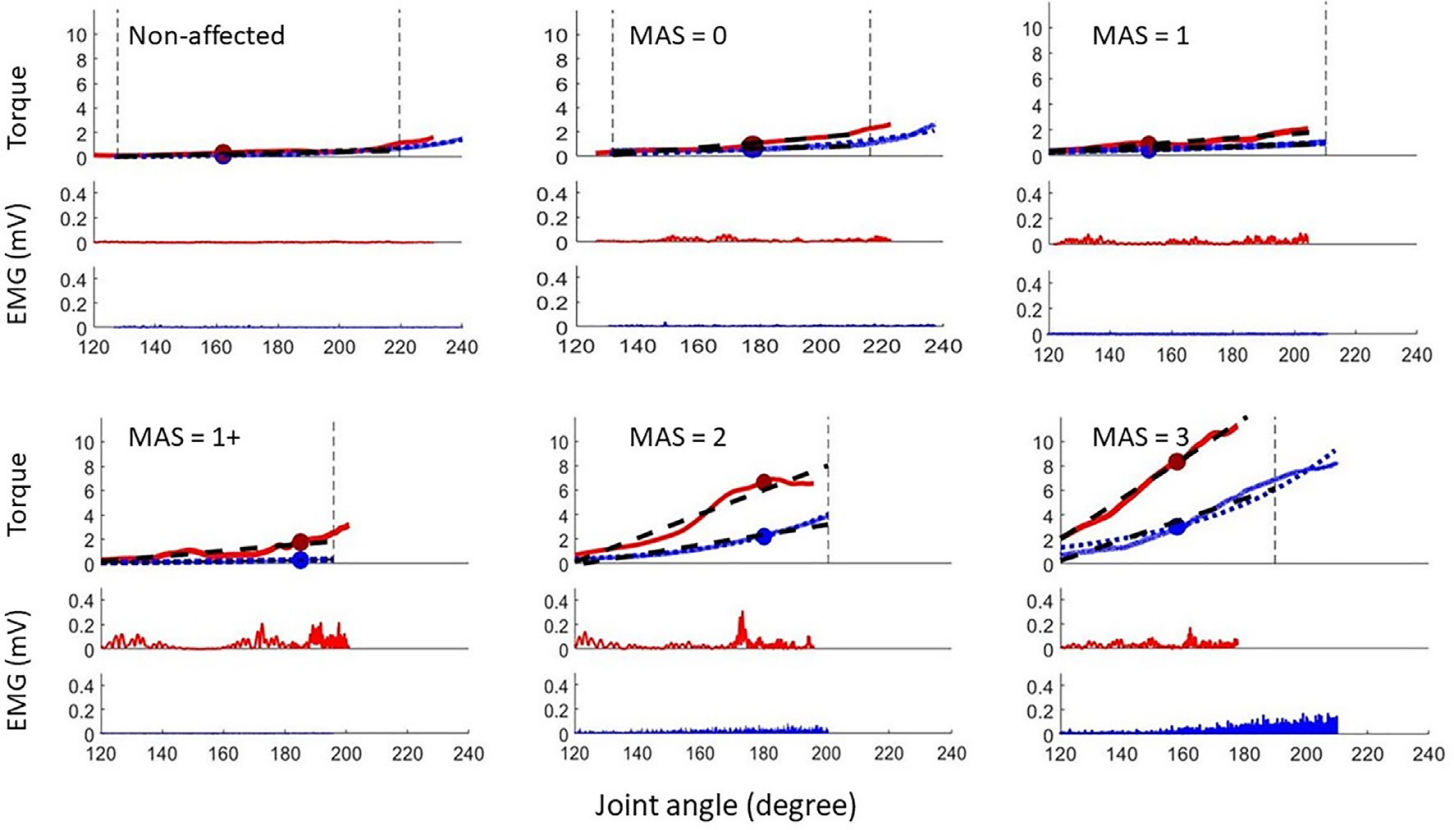
**Examples of raw data collected from stroke patients with different modified Ashworth scale (MAS).** The figure shows torque and EMG data for one slow and one fast stretch in the non-affected side in one subject and in five representative patients who scored 0, 1, 1+, 2, 3 on MAS. **The upper panels** in each block are the torque data: fast (red) and slow (blue). The vertical dashed lines mark the middle 20–80% of the range of motion used for the analysis. The dotted blue line represents the exponential function fitted to the slow torque data. The black dashed lines represent the linear function fitted to the slow and fast torque data. The **lower panel** shows the flexor carpi radialis (FCR) EMG activity during fast (red) and slow (blue) stretches. Abbreviations: MAS: modified Ashworth scale; mV: millivolt. (For interpretation of the references to color in this figure legend, the reader is referred to the web version of this article.)

In order to test whether the device is able to differentiate between patients stratified according to the MAS, we ran a one-way analysis of variance (ANOVA) to test the effect of (GROUP) i.e., the 5 different MAS scores (0, 1, 1+, 2, 3) on the different parameters extracted from the PSAD. To appreciate the differences between the data recorded from patients with different MAS scores, Fig. 4 shows examples of raw torque and EMG data acquired from the unaffected side of one patient and from five different representative patients who scored 0, 1, 1+, 2, and 3 on the MAS.

**Fig. 5:**
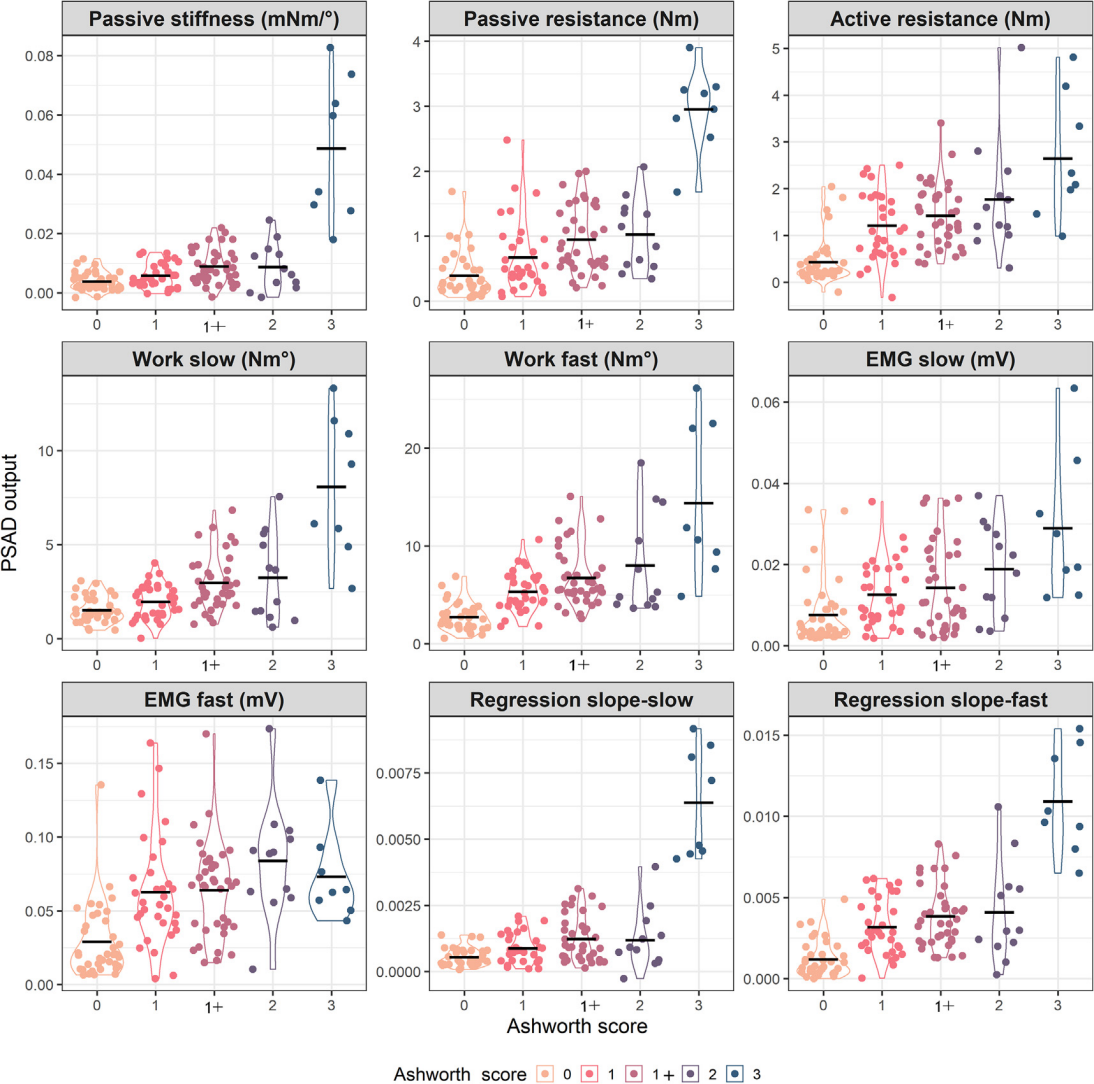
**Violin plots of the parameters extracted from the PSAD device for each MAS score.** Each panel represents a different parameter and the MAS categories are color-coded. Every dot on the scatter plot represents an individual subject during a single session. Please note that a small jitter has been applied to the individual data points to improve visualization of the spread. The black horizontal lines represent group means. Abbreviations: PSAD; Portable spasticity assessment device; MAS: modified Ashworth scale; mNm/°: milliNewtonmeter per degree; Nm: Newtonmeter; Nm°: Newtonmeter.degree; mV: millivolt.

The results of the ANOVAs showed a significant effect for GROUP on each of the PSAD parameters. Table 3 shows the F statistic values and the corresponding effect sizes (reported as partial eta squared) for each of the parameters. The test was significant at the level p <.001 for all the parameters. It is of interest to see that the parameters that varied most greatly between the groups were those related to the passive component (the slope of the torque angle function during slow stretches and the passive stiffness component). Fig. 5 is a scatter plot of the data of individual subjects/sessions with superimposed violin plots to show the spread and the distribution of the recorded data within and between the different MAS scores. The difference between the individual groups (post hoc analysis) is provided in Appendix Table A1.

**Table 3:** The effect of MAS score on the parameters extracted from the PSAD (results of analyses of variance).

**PSAD parameter**	**F_(df)_**	**Effect size** **Partial eta squared**	**Significance (p)**
EMG slow	8.80_(4,121)_	0.226	<0.001
Work slow	33.3_(4,121)_	0.524	<0.001
Slope slow	84.1_(4,121)_	0.736	<0.001
Slope fast	44.7_(4,121)_	0.597	<0.001
EMG fast	10.5_(4,121)_	0.257	<0.001
Work fast	24.9_(4,121)_	0.452	<0.001
Passive stiffness	61.2_(4,121)_	0.669	<0.001
Active resistance	19.0_(4,121)_	0.386	<0.001

PSAD: portable spasticity assessment device; MAS: modified Ashworth scale, df: degrees of freedom (model degrees of freedom (k, n-k).

### 4.4 The contribution of passive vs. Active resistance to the total measured stiffness in different MAS groups

**Fig. 6:**
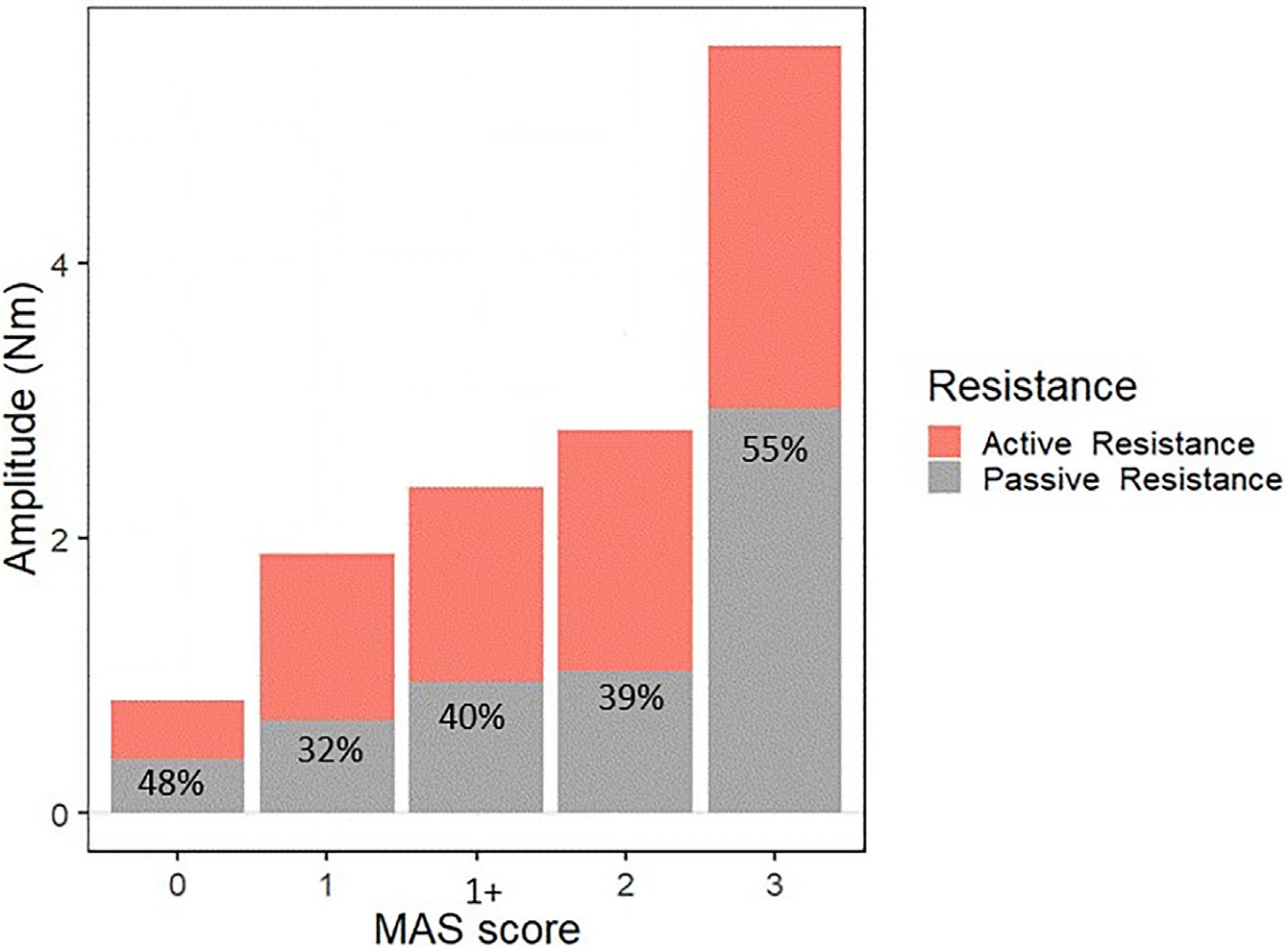
Contribution of the passive and active components to the total measured resistance in different MAS groups. Nm: Newton meter, MAS: modified Ashworth scale.

In order to test whether the passive and active components contributed to a different extent to the total resistance measured in different MAS scores, we calculated the ratio of the passive resistance measured during slow stretches to the total resistance measured during fast stretches (which includes both passive and active components). The contribution of passive resistance to the total measured torque was more than 50% in MAS = 3. On the other hand, the stretch reflex-mediated component accounts for over 50% of the torque in all other MAS grades (Fig. 6). We ran a one-way ANOVA to test the effect of the MAS scores on the ratio. The ANOVA revealed a non-significant trend towards a difference between the MAS scores (F_1,4_ = 2.35, p = 0.056).

### 4.5 Test-retest (intra-rater) reliability:

For evaluating the test–retest repeatability, we ran an intraclass correlation coefficient (ICC) analysis of absolute agreement between the two measurements performed by a single experimenter. The analysis revealed an excellent test–retest repeatability for each of the extracted parameters. The ICC (r) values were the following: for passive stiffness ICC = 0.93; active resistance ICC = 0.92; average EMG ICC = 0.95 for both slow and fast stretches; work slow ICC = 0.95; work fast ICC = 0.96; slope of the linear function fitted to the torque angle data ICC = 0.98 for slow stretches and ICC = 0.99 for fast stretches.

### 4.6 Standard error of measurement (SEm) and minimal detectable change (MDC):

The standard error of measurement was calculated based on the standard deviations of the two measurements performed by the same experimenter and the correlation between them as per the intraclass correlation coefficient analysis. When the size of the measurement error is small in reference to the mean, the minimum change that we can detect with the device becomes smaller. In our findings, the measurement error was small and the minimum detectable change at 95% confidence interval level was smaller than the mean value of each of the extracted parameters, except for passive stiffness (Table 4).

**Table 4:** Mean values, standard error of measurement (SEm) and minimal detectable change (MDC) at 68% and 95 % confidence intervals for the PSAD parameters.

Parameter	Mean	SEm	MDC68	MDC95
Work slow (Nm°)	1.976	0.443	0.626	0.868
Work fast (Nm°)	4.190	0.792	1.120	1.552
Slope slow	0.001	0.000	0.000	0.000
Slope fast	0.002	0.000	0.000	0.001
Passive stiffness (mNm/°)	0.006	0.003	0.004	0.006
Passive resistance (Nm)	0.613	0.234	0.330	0.458
Active resistance (Nm)	0.826	0.189	0.268	0.371
EMG Slow (mV)	0.010	0.002	0.003	0.004
EMG Fast (mV)	0.040	0.007	0.010	0.014

Abbreviations: SEm: standard error of measurement; MDC 68,95 is minimal detectable change at 68 and 95% confidence intervals, respectively, Nm°: Newtonmeter.degree; mNm/°: milliNewtonmeter per degree; Nm: Newtonmeter; mV: millivolt.

## 5 Discussion

The development of devices that provide accurate measurements of parameters related to spasticity is necessary to progress our understanding of the physiological mechanisms underlying it, along with the effects of different interventions which target or influence it. The purpose of this study was to test the application of a portable hand-held device to measure the active and passive components of resistance to externally-induced wrist extension in a sample of chronic stroke patients. Other devices have also been developed for this purpose including the Neuroflexor (Aggero MedTech, Alta, Sweden), a motorized device that stretches the wrist and finger flexor muscles and analyzes the measured forces using a computerized biomechanical model of the hand (Andringa et al., 2019b, Gäverth et al., 2013, Gäverth et al., 2014, Pennati et al., 2016).

There are pros and cons to the use of hand-held vs. motorized devices. Here, we shortly consider some of the differences that are specific to these two devices: (1) Motorized devices allow the application of highly controlled perturbations with constant velocities and very brief acceleration and deceleration phases. They may also be more suitable for reliable detection of stretch reflex thresholds. In hand-held devices, the control of the velocity and movement path is limited by the limitations of the human user. Due to safety concerns, however, the accurate motorized perturbations must be limited to small parts of the range of motion, which makes them less comparable to movements applied by clinicians. (2) The flexible attachment of the hand-held device to joint-specific orthoses makes it quite versatile, with the possibility of application to multiple joints and the portability makes it easy to transport to different locations. (3) When using the Neuroflexor, the fingers are placed on the moving plate in full extension which might be a problem for many spastic patients with shortening in their finger flexors. Stretching the fingers fully can not only be painful and difficult but also significantly reduce the wrist extension range of motion. (4) The active component calculated by the software which accompanies the Neuroflexor is inferred based on a model, developed and validated by the authors (Gäverth et al., 2013, Lindberg et al., 2011) without the need for EMG assessment, which may be practical. It has limitations however, including the inability to monitor whether the subject is sufficiently relaxed prior to the movement, and the inability to calculate the exact amplitude of the stretch reflex itself.

All the tools and devices that have made progress in their technical development to the stage of being used and tested by different groups, are collectively contributing to the process of developing a gold standard for measuring spasticity. Since this is the first time that the PSAD was applied to the wrist joint, we examined aspects of validity, reliability, and clinical discrimination. We also quantified the measurement error and the minimum change detectable by the device, aspects which are necessary to consider when evaluating the therapeutic effects of any rehabilitative intervention.

### 5.1 Differentiating the affected and unaffected sides:

The ability to distinguish the affected and unaffected sides is a necessary requirement for establishing the validity of the assessment device. Except the range of motion (ROM), all extracted parameters were significantly different between the two sides. The finding that the ROM is not limited on the affected side is not surprising. Many of our patients scored 0 or 1 on MAS and very few had limitations in the ROM.

The active (reflex-mediated) resistance, i.e., spasticity was significantly different between the affected and unaffected sides. Indeed, reflex hyperexcitability seems to play a significant role in the resistance to muscle stretch in stroke patients. This finding is different to what has been reported in subjects with cerebral palsy or multiple sclerosis, where the reflex-mediated stiffness seems to be larger than in control subjects only in few cases (Lorentzen et al., 2012, Willerslev-Olsen et al., 2013, Yamaguchi et al., 2018). The subjects in the study of Yamaguchi et al. also showed significantly reduced ROM and increased passive stiffness. These different findings indicate that the mechanisms underlying the increased resistance to muscle stretch differ among the different neurological conditions. In cerebral palsy, for example the increased muscle stiffness is related, among other things, to the impaired muscle growth (Willerslev-Olsen et al., 2018), which is different in the case of stroke patients who have fully developed muscles.

We also found in two of the extracted parameters a statistically significant difference between the unaffected side and the affected side with a MAS score of 0. This reflects the objective measure’s ability to detect subtle changes in resistance to the stretch that are not detected by the clinician. Similar findings were reported by Andringa et al. (2019b) using the Neuroflexor, where the values of the neural and non-neural components were significantly different between patients with a MAS score of 0 and healthy subjects. Malhotra et al. (2008) also reported detecting spastic muscle activity in subjects not identified as having spasticity using the clinical scale.

### 5.2 General agreement with the MAS

Our results showed that the extracted parameters correlate significantly with the MAS. In their review Platz et al. (2005) reported similar findings of a moderate association between the reflex-mediated EMG parameters (Fu-Mei and Mohamed, 1999, Pisano et al., 2000, Sköld et al., 1998) and a stronger association with objective measures of resistance to passive movement (Katz et al., 1992, McCrea et al., 2003, Shaw et al., 1999). Similar findings have been reported in the wrist joint using the Neuroflexor, where both the neural and non-neural components were moderately positively associated with the MAS. The authors considered the findings to attest the inadequacy of the manual test rather than highlight the construct validity of the objective tool. However, it has been considered a requirement that the obtained measures at least parallel the most used clinical test (Katz et al., 1992). It would indeed be quite problematic if the outcome measures did not correlate with the clinical test, which remains the yardstick against which we evaluate the validity of other measures (Pisano et al., 2000).

In particular, the calculated parameter *work* during fast stretches correlates strongly with the MAS and shows a gradual increase when the MAS score is higher (Fig. 6). This is a reasonable finding as *work* includes both reflex-mediated and passive components indiscriminately, similar to the MAS. From the experimenter’s perspective, *work* is the effort they need to make in order to perform the movement, and this perceived effort is what is reported when performing MAS. This is consistent with the previously described results of the difference between the unaffected side and the affected side of patients who scored 0 on the MAS. The parameter *work* was not different between these two groups, as the experimenter did not perceive any effort in performing the movement even though there were subtle changes in muscle activity and passive stiffness detected by the device.

### 5.3 Differentiating MAS scores (clinical discrimination)

The results showed that the extracted parameters were significantly different between the patients stratified by the MAS. This means that this assessment method satisfies the clinical differentiation requirement. Indeed, as more data is collected with the device, new criteria would emerge based on large datasets, both in healthy individuals and in different neurological conditions, which would enable the clinician to make decisions based on how an individual patient’s readings compare to a reference dataset.

### 5.4 Intra-rater reliability

The analysis showed that intra-rater reliability is excellent (Portney and Watkins, 2009) for each of the extracted parameters, with ICC values between 0.92 and 0.99. The motorized device for measurement of passive stiffness in the wrist and finger muscles, the Neuroflexor, showed similar test–retest reliability for the neural and non-neural components (Andringa et al., 2019b, Gäverth et al., 2013). The ability to extract such levels of consistency without using a motorized device is very promising for the future of assessment of resistance to passive movement. It is quite important to establish inter-rater reliability for wider clinical application of the device. For our purposes, however, and that is establishing the reliability of the measurement to test the effects of rTMS therapy on spasticity, the intra-rater reliability established in this study is sufficient, since the same experimenter performed all measurements prior to and following the intervention.

### 5.5 EMG during fast and slow movements

It is interesting to notice that the great increase in stretch reflex torque in the MAS = 3 group (Fig. 5) was not paralleled by an increase in EMG activity. This may be explained by the increased efficiency of torque production in muscles that lost some of their length (Gracies, 2005b). Dietz et al. (1991) also described this phenomenon in the elbow joint and concluded that secondary to the mechanics, properties of the spastic muscle change in such a way that the muscle develops more tension when it is stretched.

One can also see from Fig. 5 that there is an increase in the amplitude of the EMG during slow movements in higher MAS scores. EMG during slow movements includes the component that represents continuous descending cortical drive, i.e., spastic dystonia, defined as “tonic, chronic, involuntary muscle contraction in the absence of any stretch or any voluntary command (Gracies, 2005a, Gracies, 2005b)”. This activity often increases with the stretch even at velocities below 20°/s (Fig. A1.1) (Forman et al., 2019).

The presence of this spontaneous, although stretch-sensitive, tonic contraction during slow stretches in some of our patients means that the component of passive stiffness could be contaminated with muscle activity. In cases when the assessor is interested in quantifying the passive stiffness per se, this component might be problematic. However, since we are usually interested in evaluating the reflex-mediated stiffness for which we have possible treatments (e.g., botulinum toxin or rTMS), the assessment is still useful. It is unlikely that the presence of this activity might be caused by the patient’s non-compliance with the experimenter’s request to relax. The patients successfully relaxed their unaffected side (evident by absence of this muscle activity on the unaffected side) or even the affected side in patients with lower MAS grades (0, 1, some 1+). The EMG during slow movements was primarily present in the higher MAS scores: some 1+, most of 2 and all of 3. This component could also represent what has been described by Malhotra et al. (2008) as a position-dependent muscle activation, indeed they described five EMG firing activity patterns based on the response to position and velocity. The position-dependency of this component is highlighted by the increased EMG amplitude towards the end of the range of motion (Fig. 4).

This phenomenon is unlikely to be a problem particular to our measurement or analysis. Difficulties to isolate passive stiffness components have been reported before by Pisano et al. (2000) who also noted the occurrence of tonic EMG activity in most spastic patients. This finding confirms the prevalence of this symptom that has been largely ignored. Trompetto et al. (2019) also reported the presence of spastic dystonia in 74% of hypertonic wrist flexors in stroke subjects. At higher levels of resistance to passive movement, it becomes more and more difficult to distinguish active and passive components.

### 5.6 Standard error of measurement and minimum detectable change

When monitoring the effect of a treatment in an individual patient, a decrease of at least the size of the measurement error has to be achieved by the intervention to be interpreted as a real treatment effect. In our results, the minimal detectable difference to indicate real change at 95% confidence interval was consistently less than the mean value of each of the measures, except for the passive stiffness and passive resistance. This is indeed the first time that such a measurement has been applied to the wrist joint with such a small measurement error. In their assessment of the Neuroflexor, Andringa et al. (2019b) reported that the measurement error for all the components was large compared to the median values (70–140% of the median). This finding suggests that the PSAD would be useful to detect changes within an individual patient over time and as a response to intervention.

### 5.7 Contribution of the passive vs. Active component to the total measured resistance

The non-neurologic component (increase in muscle and soft tissue stiffness) accounts for more than 50% of the total measured resistance in patients with MAS score of 3, and reaches over 75% for some subjects in that group. In addition, the post hoc analysis showed that group MAS = 3 has a significantly higher passive stiffness and passive resistance than all other groups (Appendix Table A1.1). This does not apply to the active resistance component or EMG during fast stretches. To our knowledge, this is the first study to consider the contribution of the different components to the total measured stiffness in patients with different MAS scores. This ratio has important clinical consequences, since it suggests that stroke patients who suffer from very high degrees of resistance to passive stretching as measured with the MAS, might benefit less from any hyper-reflexia based therapy, because the change in muscle mechanics plays a larger role in the total resistance than muscle activity does. So, what has been described before, in terms of the significant contribution of the soft tissue changes and muscle contracture to the resistance to passive movement (Vattanasilp et al., 2000), may be particularly true for MAS = 3 and by definition to MAS = 4 in stroke. Having said that, it is important to consider that the muscle activity in the higher MAS grades is still very high, and as discussed above, contributes to the measurements even during slow stretches.

The finding that the active component was responsible for more than half of the total resistance for MAS scores 1, 1+ and 2, confirms the importance of the contribution of the hyperexcitability of the muscles to the total resistance to muscle stretch in stroke patients, which might be different to other neurological conditions. Having access to these values and readings will help us to understand the physiology of increased resistance to muscle stretch across different conditions.

## 6 Conclusions

We used a hand-held dynamometer which combines biomechanical and electrophysiological measures to objectively measure the resistance to externally induced joint movement in the wrist joint and discriminate components of stretch reflex and passive resistance. We demonstrated here in chronic stroke patients that the measurement is valid, in general agreement with clinical test-the MAS, shows excellent test–retest reliability and a small measurement error. These findings support the usefulness of the application to measure possible changes in the resistance to muscle stretch in the wrist joint after a therapeutic intervention.

## Declaration of Competing Interest

The authors declare the following financial interests/personal relationships which may be considered as potential competing interests: ‘One of the co-authors, Morten Haugland, works for Movotec, the company which currently owns the PSAD device. He contributed to the project by designing and testing the hand orthosis. He also adapted the software to ensure that the calculated parameters were correct for the novel application to the wrist. He did not, however, participate in the experiments with the patients nor did he interfere with the design of the study, the statistical analysis or the interpretation of the results. None of the other authors declares a conflict of interest.’.
