# Supplementary material for: Measuring resistance to externally induced movement of the wrist joint in chronic stroke patients using an objective hand-held dynamometer
Source: Clin Neurophysiol Pract. 2023 May 16;8:97–110. doi: 10.1016/j.cnp.2023.05.001 (PMC10238875; doi:10.1016/j.cnp.2023.05.001)
Supplement: Supplementary data 1 [file mmc1.docx]

**Appendix A1**


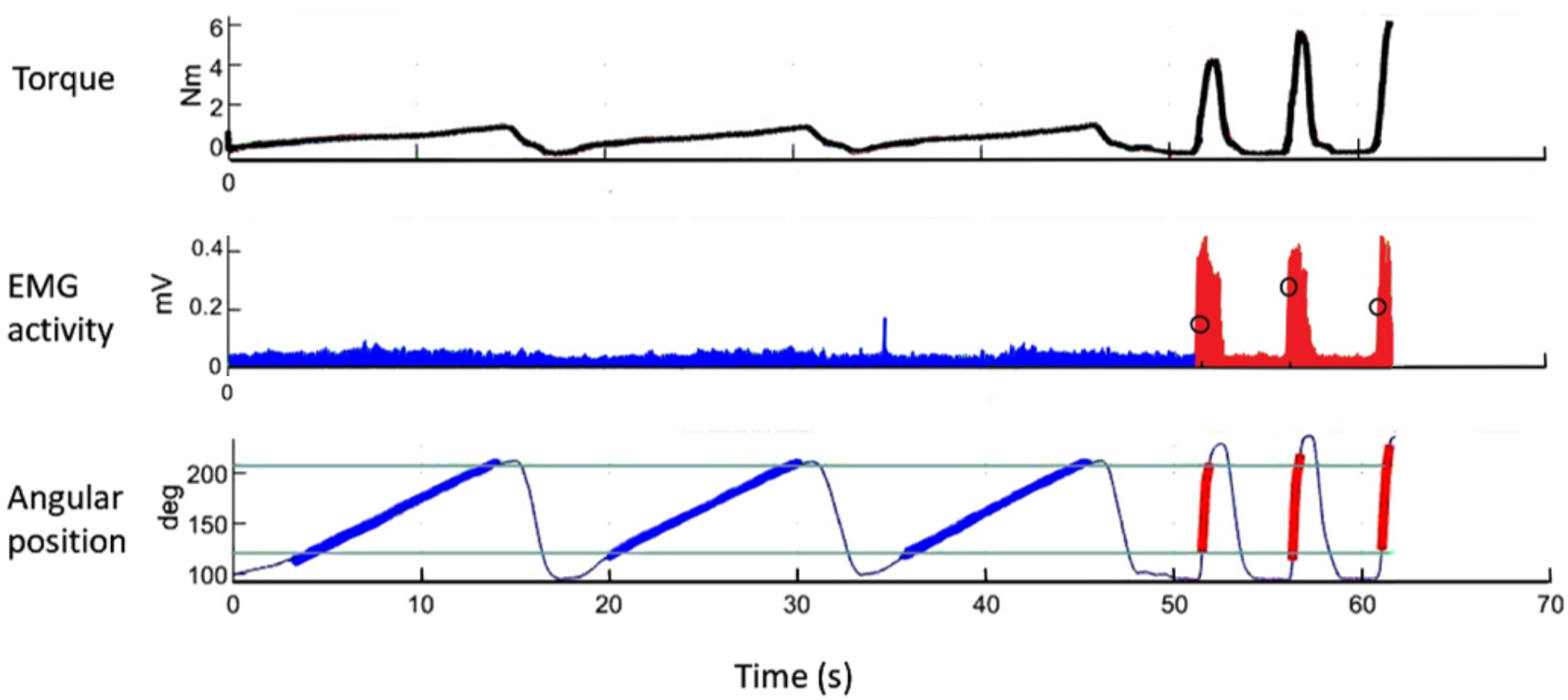


**Figure A1.1.** Example raw PSAD data: **angular position (bottom panel)**, the wrist extension movement starts from a position of full flexion (~100°, where at 90° the hand will be at right angles with the forearm) until the end of the available ROM. Slow (blue) and fast (red) stretches are detected by the software. **FCR EMG** (middle panel): Rectified and band-passed filtered EMG collected during slow movements is marked with blue and that during fast movement is marked with red. Notice the low- level background EMG during slow movements which slightly increases with stretching. The circles on the EMG during fast movements are the EMG peaks that mark the reflex-mediated response (stretch reflex). **Torque (top panel)** is the sum of the applied torque, weight and inertia.

**Table A1.1.** Post hoc results to compare the different PSAD outcome parameters among subjects with different modified Ashworth scale (MAS) scores (Games Howell correction for multiple comparisons).

| **Outcome measure** | **MAS score** | **compared to** | **Significance (p)** |
| --- | --- | --- | --- |
| EMG slow | MAS 0 | MAS 1 | 0.086 |
|  |  | MAS1+ | 0.034 |
|  |  | MAS 2 | 0.037 |
|  |  | MAS 3 | 0.064 |
| EMG fast | MAS 0 | MAS 1 | <0.001 |
|  |  | MAS1+ | <0.001 |
|  |  | MAS 2 | 0.003 |
|  |  | MAS 3 | 0.025 |
| Work slow | MAS 0 | MAS 1 | 0.177 |
|  |  | MAS1+ | <0.001 |
|  |  | MAS 2 | 0.145 |
|  |  | MAS 3 | 0.009 |
| Work fast | MAS 0 | MAS 1 | <0.001 |
|  |  | MAS1+ | <0.001 |
|  |  | MAS 2 | 0.034 |
|  |  | MAS 3 | 0.025 |
| Slope slow | MAS 0 | MAS 1 | 0.029 |
|  |  | MAS1+ | <0.001 |
|  |  | MAS 2 | 0.354 |
|  |  | MAS 3 | <0.001 |
| Slope fast | MAS 0 | MAS 1 | <0.001 |
|  |  | MAS1+ | <0.001 |
|  |  | MAS 2 | 0.048 |
|  |  | MAS 3 | <0.001 |
| Passive stiffness | MAS 0 | MAS 1 | 0.185 |
|  |  | MAS1+ | <0.001 |
|  |  | MAS 2 | 0.287 |
|  |  | MAS 3 | 0.007 |
| Passive resistance | MAS 0 | MAS 1 | 0.109 |
|  |  | MAS1+ | <0.001 |
|  |  | MAS 2 | 0.015 |
|  |  | MAS 3 | <0.001 |
| Active resistance | MAS 0 | MAS 1 | <0.001 |
|  |  | MAS1+ | <0.001 |
|  |  | MAS 2 | 0.021 |
|  |  | MAS 3 | 0.013 |

**Appendix A2. Principal factor analysis to reduce the number of outcome parameters considered for the clinical application**

**A2.1 Rationale and analysis methods**

In this study, we extracted multiple outcome parameters from the data collected by the dynamometer. This was done in compliance with the recommendation of the European Consensus on the Concepts and Measurements of the Pathophysiological Neuromuscular Responses to Passive Muscle Stretch (van den Noort et al., 2017). Additionally, we were interested in describing the wrist joint’s resistance to passive stretch in detail in order to distinguish the different components and the way they interact with each other and change with the increased severity of resistance. However, extracting as many outcome parameters as we did here may be impractical for the clinical application of the device as it makes the interpretation of the extracted data more difficult. We thus wanted to reduce the number of outcome measures used in future studies by extracting the smallest number of underlying factors that explain the maximum amount of variability in the data.

**A2.2 Principal factor analysis**

We subjected the 10 parameters extracted from the PSAD data to a factor analysis with orthogonal rotation (Varimax). We first tested if our sample and correlation structure were adequate for a factor analysis. The Kaiser- Meyer-Olkin (KMO) measure verified the sampling adequacy for the analysis with a KMO value of 0.78 and the Bartlett’s test of sphericity resulted in a $x^{2}$ value of 2822, p<0.001, indicating that the correlation structure is adequate for factor analysis. To reach at the smallest number of factors which underlie the variability in the data, we used principal axis factoring method with a cut off of 0.5 and the Kaiser’s criterion of eigenvalues greater than 1 (Field, 2013).

**A2.3 Results of principal factor analysis**

The analysis yielded a solution with three primary factors which account for 86% of the variability in the data. The result of the factor analysis is shown in Table A2.1. The degree to which each variable correlates to an underlying factor is called factor loading, a value between 0-1 that indicates the degree of variance explained by the variable on a specific factor. as a rule of thumb, 0.7 or higher factor loading represents that the factor extracts sufficient variance from that variable.

**Table A2.1.** Factor loading table.

| **Parameter** | **Factor1** | **Factor2** | **Factor3** |
| --- | --- | --- | --- |
| Slope slow | .933 |  |  |
| Passive stiffness | .919 |  |  |
| Work slow | .777 |  |  |
| Passive resistance | .711 |  |  |
| Active resistance |  | .922 |  |
| Slope fast |  | .880 |  |
| Work fast |  | .768 |  |
| EMG fast |  |  | .959 |
| Number of stretch reflexes |  |  | .767 |
| EMG slow |  |  | .595 |

*Loading factors less than 0.5 have been suppressed.

**A2.4 Discussion** **and conclusion**

To reduce the complexity of the data extracted from the PSAD device, we ran a principal factor analysis with the aim of extracting the minimum number of factors that explain the maximum variability in the data. By looking at which variables have high factor loading to each of the underlying factors in our analysis, we may conclude that factor 1 represents the passive component, factor 2 represents the stretch reflex component and factor 3 represents muscle activity (stretch reflex and spastic dystonia). In the future, it would be possible to integrate this principal analysis in the analysis software to readily provide for clinicians a summary of these three components.
